# Meningioma-Related Epilepsy: A Happy Ending?

**DOI:** 10.3390/jpm13071124

**Published:** 2023-07-11

**Authors:** Giada Pauletto, Annacarmen Nilo, Sara Pez, Maria Elisa Zonta, Daniele Bagatto, Miriam Isola, Lorenzo Verriello, Mariarosaria Valente, Miran Skrap, Tamara Ius

**Affiliations:** 1 Neurology Unit, Head-Neck and Neurosciences Department, Santa Maria della Misericordia University Hospital, 33100 Udine, Italy; giada.pauletto@asufc.sanita.fvg.it (G.P.); lorenzo.verriello@asufc.sanita.fvg.it (L.V.); 2 Clinical Neurology Unit, Head-Neck and Neurosciences Department, Santa Maria della Misericordia University Hospital, 33100 Udine, Italy; mariarosaria.valente@uniud.it; 3 Department of Medicine, University of Udine, 33100 Udine, Italy; sara.pez91@gmail.com (S.P.);; 4 Neuroradiology Unit, Department of Diagnostic Imaging, Santa Maria della Misericordia University Hospital, 33100 Udine, Italy; daniele.bagatto@asufc.sanita.fvg.it; 5 Division of Medical Statistics, Department of Medicine, University of Udine, 33100 Udine, Italy; miriam.isola@uniud.it; 6 Neurosurgery Unit, Head-Neck and Neurosciences Department, Santa Maria della Misericordia University Hospital, 33100 Udine, Italy; miran.skrap@asufc.sanita.fvg.it (M.S.); tamara.ius@gmail.com (T.I.)

**Keywords:** epilepsy, meningioma, predictors, seizure, surgery, oncology

## Abstract

(1) Background: More than one-third of patients with meningiomas experience at least one seizure during the course of their disease, and in the 20–50% of cases, seizure represents the onset symptom. After surgery, up to 30% of patients continue to have seizures, while others may experience them later; (2) Methods: The study analyzed retrospectively the risk factors for pre-operative seizures in a large cohort of 358 patients who underwent surgery for newly diagnosed brain meningioma; (3) Results: We identified age, peritumor edema, and location as risk factors for seizure at the onset. Patients with seizures differed from patients without seizures for the following characteristics: younger average age, lower pre-operative Karnofsky Performance Status (KPS), location on the convexity, lower Simpson Grade, lower incidence of pre-operative neurological deficits, and higher incidence of pre-operative peritumor edema. After 24 months, 88.2% of patients were classified as Engel class Ia, and no correlation with disease progression was observed; (4) Conclusions: Meningioma-related epilepsy has generally a positive outcome following surgery and it seems not to be linked to disease progression, even if further studies are needed.

## 1. Introduction

Meningiomas account for 16–38% of all intracranial tumors in adults [1]. Approximately one-third of patients with meningiomas experience at least one seizure during their disease, and in 20–50% of cases, seizure is the first symptom [2,3]. Although surgical resection can offer seizure freedom in 60–90% of meningiomas, seizures continue to persist after surgery in up to 30% of patients [4]. 

Patients with meningiomas have a seizure incidence of over 33% during the course of their disease. However, meningioma-related epilepsy has received insufficient research attention, with the majority of studies focusing on tumor growth, resection size, and survival rates. It is essential to determine predictors of pre-operative seizures and post-operative seizure freedom, since epilepsy significantly impacts the patient’s quality of life [5]. Thus, achieving seizure freedom is a crucial goal after surgery for meningiomas, which can often be surgically removed and have a favorable long-term prognosis [5].

Recent studies have investigated the link between meningiomas and seizures. In this clinical setting, a wide range of seizure outcomes has been reported, mainly for different classification schemes adopted [6]. In addition, different uncertainties persist concerning the risk factors and the techniques for managing seizures in meningioma patients. 

Studying the population of patients with seizures prior to meningioma resection is crucial in comprehending this intricate disease process, since they seem to have poorer outcomes than those without pre-operative seizures [2,7,8,9]. 

The aims of the present work were threefold: firstly, determining the frequency of seizures among individuals who underwent surgery for supratentorial meningiomas; secondly, exploring the risk factors linked to pre-operative seizures; and finally, assessing long-term seizure outcome in patients with meningioma-related epilepsy.

## 2. Materials and Methods

### 2.1. Study Population

We performed a retrospective analysis of a consecutive series of patients who underwent surgical treatment for newly diagnosed intracranial meningioma at the Neurosurgical Unit of Udine University Hospital, between January 2016 and November 2020.

Patients were enrolled according to the following inclusion criteria:

- age ≥ 18 years;
- newly diagnosed meningioma cases and non-previous treatments;
- confirmed histological diagnosis of meningioma according to the updated 2016 World Health Organization (WHO) guidelines [10];
- pre- and post-operative brain Magnetic Resonance Imaging (MRI) that were either performed at our Institution or available on the picture archiving and communication system (PACS) for review.

Additionally, patients were included if they were able to undergo a standard clinical and radiological follow-up starting from the 30th day after surgery. For those cases with seizure as onset symptom, diagnosis of tumor-related epilepsy was performed according to the International League Against Epilepsy (ILAE) definition [11]. Engel Class at 12- and 24-month follow-up was used to compute post-operative seizure outcome [12].

Patients were excluded in case of incomplete or inaccurate data in clinical, radiological, and surgical records, or if they were lost to follow-up. We only included patients who underwent a surgical procedure aimed at total or subtotal resection of the lesions. We excluded those who underwent biopsies.

### 2.2. Clinical Data

Clinical information was retrieved from medical records.

We collected the following data: gender, age, onset symptom, tumor localization and side, seizure type and frequency, type and number of anti-seizure medications (ASMs), pre-operative electroencephalogram (EEG), extent of resection (EOR), and post-operative seizure outcome.

We considered as focal neurological deficits: disorders affecting body movement and sensitivity, and cranial nerves. Regarding onset symptoms, we took into account, beyond seizures, other symptoms like dizziness, altered mental status, memory loss, intractable headaches, and incidental findings.

Tumor locations were categorized as skull base (sphenoid ridge, suprasellar, posterior fossa, olfactory groove, middle fossa/Meckel cave, and tentorial meningioma) or non-skull base (parasagittal, falcine, convexity, and lateral ventricular meningioma).

Extent of resection was considered as gross-total for Simpson I–III resection (or with no signs of tumor remnant on post-operative MRI) and sub-total for Simpson IV–V (or with obvious tumor remnant on post-operative MRI) [13]. Surgical complications such as hemorrhage, central nervous system (CNS) or wound infection, and cerebrospinal fluid (CSF) leakage were recorded.

The 2017 ILAE classification [14] was applied to classify seizures. Seizure frequency was assessed before surgery and after surgery every 6 months for 2 years.

Engel Class [12] at last follow-up was retrospectively assigned on the basis of self-completed seizure diaries and further grouped into favorable (class Ia) and unfavorable (class > Ia) outcomes.

Patients without reliable seizure outcome data and a minimum of 6-month postoperative follow-up were excluded.

### 2.3. Imaging Analysis and Region of Interest (ROI)-Drawing Process

All enrolled patients underwent a pre-operative brain MRI scan (Achieva 3 Tesla Scanner, Philips Medical Systems, Best, The Netherlands or Aera 1.5 Tesla Scanner, Siemens, Erlangen, Germany). The radiological evaluation included identifying the location of lesion, the presence of multiple meningiomas and/or meningiomatosis, the involvement of the subtentorial location, tumor diameter (measured in centimeters), and tumoral volume (measured in cubic centimeters). This was accomplished using isotropic volumetric T1-weighted sequences before and after intravenous administration of a paramagnetic contrast agent (gadolinium). Additionally, turbo spin echo (TSE) T2-weighted and fluid-attenuated inversion recovery (FLAIR) T2-weighted sequences were used to determine the edema volumes (measured in cubic centimeters) prior to anti-edema therapy.

To calculate the volume of the contrast-enhanced lesion and edema, a region of interest (ROI) was drawn in a volumetric enhancing postcontrast T1-weighted sequence (Mprage sequence) and in a T2-weighted TSE or FLAIR axial sequences using the software Horos. In 3D T1-weighted contrast-enhanced sequences, a pencil-draw semi-automatic tool was used to outline the lesion on all subsequent slices with a minimum thickness of 1.00 mm, conforming to the margins of the contrast-enhanced lesion with the software Horos. The same method was applied using T2-weighted images to outline the hyperintensity area, which is defined as “the high signal on T2-weighted imaging of brain sequences”, referring to the entire volume including the tumor lesion and associated tumor mass to obtain a new ROI.

**Figure 1:**
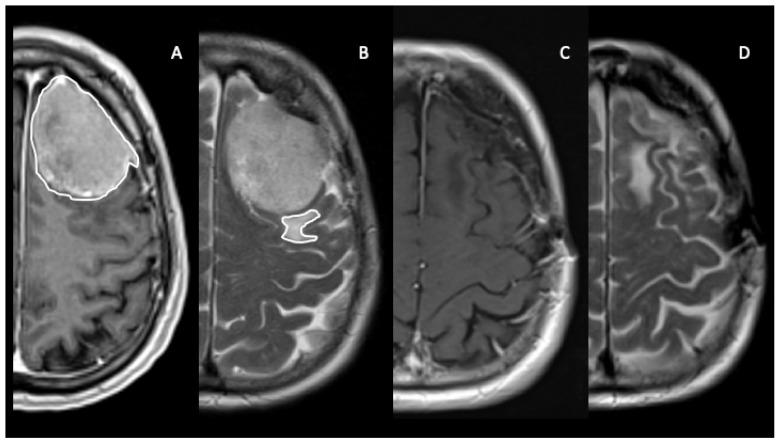
A brain MRI of a patient with left frontal meningioma. (**A**,**B**) show pre-operative postcontrast T1-weighted (**A**) and T2-weighted TSE MRI sequences, demonstrating ROI segmentation of lesion and peritumor edema, circumferentially outlined, using Horos software. (**C**,**D**) display postcontrast T1-weighted and T2-weighted TSE MRI sequences after surgical resection.

Figure 1 summarizes one exemplificative case of meningioma with edema.

### 2.4. Surgery

The patients underwent several transcranial and skull base approaches according to the site of the meningioma. For lesions located in the olfactory groove and anterior floor different approaches were used, including supraorbital, cranio-orbital, and bi-frontal approaches. Meningiomas situated in the spheno-orbital and temporal floor were removed using the cranio-orbital zygomatic approach, while spheno-petroclival meningiomas were eliminated through retro-sigmoid one. Tentorial meningiomas were removed using the suboccipital and/or retro-sigmoid approaches [15].

The surgical resections of large meningiomas, especially when encasing nerves and/or the main cerebral vascular trunks and/or their perforating vessels skull base, were planned with the assistance of intra-operative neurophysiological monitoring.

For challenging cases, to determine whether adjunctive embolization of feeders was feasible [16,17], all patients underwent evaluation by a neuroradiologist.

### 2.5. Statistical Analysis

Categorical variables were reported as percentages, continuous variables were reported as mean ± standard deviation or median and range as appropriate, according to the data distribution. Normality of the continuous variables was tested using the Shapiro–Wilk test. The *t*-test or Mann–Whitney U-test, as appropriate, was used to compare continuous variables between groups (patients with pre-operative seizures versus patients without pre-operative seizures). Comparisons between nominal and categorical variables were made with a Chi-square test or Fisher’s exact test. For the outcome analysis, Engel classification was dichotomized as Engel Class Ia versus Engel Class > Ia (Ib-IV) (patients were either completely seizure free or not completely seizure free). A uni- and multi-variate ANOVA analysis along with contrast analysis and post hoc tests was performed to identify risk factors influencing pre-operative seizure onset and post-operative seizure outcome.

Continuous variable correlations were investigated with Pearson’s bi-variate correlation. All two-tailed statistical significance levels were set at *p* < 0.05.

All analyses were conducted using Stata/SE (StataCorp. 2015. Stata Statistical Software: Release 14. College Station, TX, USA: StataCorp LP) for Mac.

## 3. Results

### 3.1. Study Population and Characteristics of Meningioma-Related Epilepsy

Among 422 patients who underwent surgery for meningioma in the study period, 358 were enrolled. The neuroradiological diagnosis was consistently reached within a timeframe of two months from the initial onset of symptoms. The median time between diagnosis and surgery was 4 months (range 2–7 months). Only twelve patients (3.3%) underwent post-operative radiotherapy.

Demographic, clinical, histological, molecular, and radiological data of patients included in the study are summarized in Table 1.

Seizure was the onset symptom in 73 cases (20.4%). In detail, 45 subjects (61.6%) presented a single seizure, while the remaining 28 (38.4%) had more seizures before diagnosis. Focal seizures were detected in 28 cases (38.4%), while those with focal onset evolving into bilateral tonic-clonic seizures were 45 (61.6%). Seizures semiology was as follows: motor seizures in 79.5% of patients and non-motor in the remaining 20.5% (13.7% somato-sensitive, 2.7% cognitive, 2.7% autonomic, and 1.4% affective, respectively).

All patients were under ASMs: particularly, 67 (91.7%) were in monotherapy, while only six (8.3%) were in polytherapy. Levetiracetam was the most common ASM used in monotherapy (82.1%, *n* = 55), followed by Carbamazepine (10.4%, *n* = 7) and Valproic Acid (7.5%, *n* = 5). In polytherapy schemes, Perampanel was prescribed as first add-on drug in all patients.

Table 2 reports the main characteristics of patients with meningioma-related epilepsy.

A comparative analysis between the subgroup of patients with seizure onset (20.4%) and the one without seizure onset (79.6%) was performed to investigate the pre-operative differences influencing the clinical onset (see Table 3).

Age (OR 0.97, 95% CI 0.95–0.99, *p* = 0.003), pre-operative neurological deficit (OR 0.51, 95% CI 0.30–0.87, *p* = 0.014), tumor location (OR 0.06, 95% CI 0.01–0.48, *p* = 0.007), the presence of peritumoral edema (OR 4.56, 95% CI 2.46–8.46, *p* < 0.001) and tumoral grade (OR 2.17, 95% CI 1.12–4.22, *p* = 0.022) resulted, at univariate analysis, risk factors for clinical onset with seizures.

Regarding tumoral grade, patients with WHO grade II meningiomas had a higher risk than those with WHO grade I meningiomas of developing pre-operative seizures (*p* = 0.022).

With regard to tumor site, an increased risk was demonstrated for convexity meningiomas (*p* < 0.007). Among non-convexity meningiomas, there was a higher risk of pre-operative seizures for middle cranial fossa (MCF) meningiomas compared with meningiomas of posterior cranial fossa (PCF) (OR 29.57, 95% CI 3.44–254.08, *p* = 0.002).

Multivariate analysis confirmed age (*p* < 0.001), peritumoral edema (*p* < 0.001), and meningioma location (*p* = 0.016) as independent risk factors for pre-operative seizures (Table 4).

Pre-operative seizures were not risk factors, either for post-operative deficits (*p* = 0.91) or for disease progression (*p* = 0.97) (Table 3).

### 3.2. Post-Surgery Seizure Outcome

Among patients with meningioma-related epilepsy, eight developed early onset seizures after surgical treatment.

Seizure outcome was assessed every six months after surgery. All patients had 12-months follow-up; at 24 months, five patients were lost, and we could retrieve data from 68 subjects.

At 12 months, 87.6% (64/73) of patients were in Engel class Ia; at 24 months, 60 patients were still seizure-free (88.2%).

Non-seizure-free patients (Engel Class > Ia) had frequently meningiomas involving midline (33.3% vs. 6.2%, *p* = 0.04) or medial temporal fossa (33.3% vs. 9.5%, *p* = 0.05). Subjects under ASM polytherapy had a higher risk of seizure persistence after surgery (22.2% vs. 1.6%, *p* = 0.03). Evaluation at 24 months confirmed the association between seizure persistence and tumor location (midline lesions, *p* = 0.04) and polytherapy (*p* = 0.04) (Table 5).

No correlations were identified between seizure outcome and: histological grade (*p* = 0.06), tumor volume (*p* = 0.35) and EOR (*p* = 0.06).

Among patients without pre-operative seizures (*n* = 285), six (2.1%) had early-onset seizures after surgery and they were not treated with ASMs. Only one patient developed focal sensory seizures, six months after surgery and, consequently, he was put under treatment.

## 4. Discussion

Predictors and outcomes of meningioma-related epilepsy remain understudied, probably because most investigations have been dealing mainly with surgical aspects and prevention of neurological deficits. However, regardless of whether or not seizures may respond to ASMs, they represent a significant cause of morbidity and poor quality of life [9,18,19]. Therefore, proper understanding of these features is imperative to preventing seizure occurrence and guiding treatment strategies for patients with intracranial meningiomas. This is especially important considering the increase in meningioma detection due to the widespread availability of various neuroimaging modalities.

In our study, frequency of pre-operative meningioma-related seizures does not differ from data reported in the literature, affecting 20.4% of the entire population. Large previous and recent studies observed a percentage between 12.9% and 31.3% [3,9,19,20,21]. Demographic, clinical and radiological characteristics of patients are also in line with the literature [2,18,19,22,23,24,25,26] (Table 6).

Younger patients seem to more frequently develop pre-operative seizures. One could speculate that it may be due to cerebral plasticity, which influences active epileptogenic processes. Harward et al. suggest that a greater incidence of aggressive meningiomas in younger subjects may be the cause of frequent pre-operative meningioma-related seizures [24]. However, this observation is mainly related to the pediatric population, who are not represented in our study. Since younger patients in our sample had an age between 30 and 50 years, another possible factor influencing the occurrence of pre-operative seizures may be the effect of peritumoral edema. A certain level of cerebral atrophy, which is present in older patients, is protective against early symptoms of edema, such as seizures.

Gender does not appear to influence seizure occurrence in our study. There are controversial data regarding gender as a risk factor for pre-operative meningioma-related seizures; in fact, some authors observed a prevalence of seizures in females [27], while others reported a prevalence in males [8,28]. Furthermore, there are also studies that did not find any association between gender and meningioma-related seizures, as in our population [19,21,27]. Our results may depend on sample size, which presents a globally equal distribution in gender. Furthermore, the majority of women in our population were in peri- and post-menopausal age, thus with a possible reduction in sex-hormone-related epileptogenic mechanisms.

Tumor location is one of the main risk factors for meningioma-related epilepsy; in fact, meningiomas nested on cerebral convexity and in the temporal lobe are more prone to ignite seizures. This correlation depends on the involvement of larger cortical surface and the intrinsic predisposition to generate seizures of central and temporal brain areas [7,29,30,31]. Tumor side does not seem to influence seizure occurrence as reported by Howard et al. [24].

Our data confirm also that peritumoral edema represents the most significant risk factor for pre-operative seizures [31]. Chaichana et al. [28] reported that the presence of peritumoral edema at pre-surgical imaging increased the possibility to develop pre-operative seizures by a factor of three. Li et al. [9] identified peritumoral edema >/= 1 cm as one of the risk factors for pre-operative seizures, as well as in Bogdanovic’s recent work [19]. Edema may enhance seizures, determining neuronal death and excitotoxicity, by mean of mass effect on cerebral cortex. Furthermore, the breakdown of the blood–brain barrier, due to neo-angiogenesis and impaired vessel permeability, may determine local inflammatory responses and glutamate release that facilitate epileptogenic firing [32].

Besides the presence of edema, meningiomas are slow-growing tumors, thus they can induce changes in peritumoral brain areas that drives epileptogenesis. Among morphologic and functional changes happening in the brain tissue adjacent to meningiomas, there are alterations in synaptic vesicles trafficking and in glial gap-junction coupling, higher concentration of voltage-dependent Na+ channel, Ca++, and glutamate receptors and loss of inhibitory GABAergic synapses [24,33]. Changes in ion and neurotransmitter concentration as well as alterations of extracellular matrix are also advocated. These mechanisms, however, are common to different brain injuries and they have been extensively investigated, especially in glioma-related epilepsy [34]. Moreover, in meningioma-related epilepsy, NF2 mutation was shown to be predictive marker for seizures, via an indirect mediation effect with atypical histology and edema [35].

In contrast with the literature [19,24,36], we observed a significant statistical correlation between the occurrence of pre-operative seizures and histological grading. Particularly, patients with WHO grade II meningiomas are at higher risk of developing seizures, maybe due to invasion of cerebral cortex and the consequent cascade of local events leading to epileptic network building up [37], in addition to the increased development of peritumoral edema [24,28].

Patients with meningioma-related epilepsy are less associated with concomitant focal neurological deficits. These findings are consistent with the work of Seyedi et al. [26] and Hwang et al. [20]. Focal neurological deficits are usually expressions of rapidly destructive pathological processes, thus limiting the organization of epileptic foci and networks. Moreover, patients with seizures at clinical onset, especially in case of motor ones, may receive earlier diagnosis and surgery, preventing the occurrence of structural damages.

Surprisingly, mean pre-operative KPS value appears to be lower among patients with meningioma-related epilepsy; however, this apparently contrasting result can be explained by the impact of seizures on quality of life and by ASMs side effects.

Long-term seizure freedom after surgery occurred in 87.5% of cases in our population. In the literature, seizure freedom rate ranges between 60 and 90%, even if follow-up periods may vary [19,35].

Predictive factors for post-operative seizure outcome, at 12 months, are tumor location and side, and medical therapy. Lesion side and medical therapy have been confirmed also at long-term follow-up. Seyedi et al. [26] reported a relation between poor seizure outcome and meningioma location in the left hemisphere. In our population, instead, midline cortical involvement is related with worse post-operative seizure control, maybe due to the development of multiple epileptic foci or easier and faster spreading of ictal discharges. Convexity meningiomas, on the contrary, are associated with a better seizure outcome, as observed in previous studies [19,27]. Surgical approach for convexity meningiomas permits, in fact, an increased probability of gross total resection, with less manipulation and minor risk of vascular accidents.

In our study, EOR does not influence post-operative seizure outcome, in contrast with the literature [24,25,35]. Also, tumoral volume, histology, pre-operative seizure type, and frequency do not appear to be related with Engel class. It is possible that these data depend mainly on sample size.

Finally, patients treated with only one ASM have a better seizure outcome than those on polytherapy. This result is expected, since a pre-surgical pharmaco-resistance indicates more complex epileptogenic phenomena and diffuse epileptic network that may persist despite surgery.

To prevent the development of pharmaco-resistance, a prompt pre-surgical treatment with a carefully chosen ASM should be established, taking into consideration side effects, especially in the case of elderly patients. ASMs with a broad spectrum of efficacy, little or no interactions and easier titration scheme should be preferred. In our population, we chose Perampanel as add-on therapy, when needed, because it is generally well tolerated and presents an easy therapeutic scheme, being administered only once a day, before sleep [38,39]. Prophylaxis with ASMs, instead, should be avoided because it does not have any proven role in preventing post-operative seizures and it may cause useless side effects [7,9,19,38,40].

Furthermore, ASMs withdrawal is often troublesome and post-resection seizures may arise from a fast pharmacological tapering [5].

**Table 6:** Literature review of studies about patients with meningioma and seizures.

**Author (year)**	Gadot et al. (2021) [35]	Baumgarten et al. (2021) [41]	Schneider et al. (2019) [25]	Hwang et al. (2019) [20]	Seyedi et al. (2018) [26]	Wirsching et al. (2016) [21]	Zheng et al. (2013) [42]
**Number of patients**	57	420	187	303	295	661 *	97
**Female** **(*n*, %)**	29/57 (51%)	282 (67%)	121 (65%)	215 (71%)	197 (66.8%)	532 (80.5%)	59 (60.8%)
**Location**	- Non-Skull Base: 42/57 (73.7%) - Skull Base: 15/57 (26.3%)	- Non-Skull Base: 273/420 (65%) - Skull Base: 147/420 (35%)	- Non-Skull Base: 134/187 (71.6%) - Skull Base: 53/187 (28.4%)	- Non-Skull Base: 199/303 (65.7%) - Skull Base: 104/303 (34.3%)	- Non-Skull Base: 208/295 (70.5%) - Skull Base: 87/295 (29.5%)	- Non-Skull Base: 320/661 (48.4%) - Skull Base: 341/661 (51.6%)	- Non-Skull Base: 81/97 (83.5%) - Skull Base: 16/97 (16.5%)
**Pathology**	- WHO I: 40/57 (70.2%) - WHO II: 14/57 (24.6%) - WHO III: 3/57 (5.2%)	- WHO I: 244/420 (58%) - WHO II: 164/420 (39%) - WHO III 7/420 (1.7%) - Unknown: 5/420 (1.3%)	- WHO I: 140/187 (75%) - WHO II–III: 47/187 (25%)	- WHO I: 251/303 (82.8%) - WHO II: 44/303 (14.5%) - WHO III: 8 (2.6%)	- WHO I: 268/295 (90.8%) - WHO II-III: 27/295 (9.2%)	- WHO I: 638/779 (81.9%) - WHO II: 119/779 (15.3%) - WHO III: 22/779 (2.8%)	- WHO I: 90/97 (92.8%) - WHO II: 6 (6.2%) - WHO III: 1 (1.0%)
**Pre-operative seizure (*n*, %)**	57/57 (100%)	87/420 (20.7%)	187 (100%)	49 (16.2%)	72/295 (24.4%)	244/779 (31.3%)	97/97 (100%)
**Seizure type**	- Focal: 30/57 (52.6%) - Focal to bilateral tonic-clonic evolution: 25/57 (43.9%) - Unknown: 2/57 (3.5%)	Unknown	- Focal: 63/187 (34%) - Focal to bilateral tonic-clonic evolution: 124/187 (66%)	Unknown	Unknown	Available in 237 patients - Focal: 114/237 (48.1%) - Focal to bilateral tonic-clonic evolution: 123/237 (51.9%)	- Focal: 45/97 (46.4%) - Focal to bilateral tonic-clonic evolution: 52/97 (53.6%)
**Seizure Outcome**	Seizure-free: 42/57 (73.7%)	Pre-operative seizures (*n* = 87): seizure-free 24/87 (72.4%)	Seizure-free: 169/187 (90%)	Pre-operative seizures (*n* = 49): seizure-free 33/49 (67.3%)	Pre-operative seizures (*n* = 72): seizure-free 46/72 (63.8%)	Pre-operative seizures (*n* = 244): seizure-free 144/244 (59%)	Seizure-free: 50/97 (51.5%)
**ASM prophylaxis**	54/57 (94.7%)	Unknown	Unknown	294/303 (97%)	57/72 (79.2%)	Unknown	58/97 (60%)
**Tumor recurrence/progression (*n*, %)**	- Residual disease: 13/57 (22.8%) - Recurrent disease: 10/57 (17.5%)	Tumor recurrence: 44/420 (10.5%)	Tumor progression: 18/187 (9.6%)	48 (15.84)	- Tumor progression: 70/295 (23.7%) - Unknown: 22/295 (7.5%)	203/779 (26.1%)	2/97 (2.1%)
**Prognostic factors related to pre-operative seizures**	/	- Temporal location - Pre-operative edema	/	- Vasogenic edema - Falx/parasagittal tumor location - Absence of pre-operative neurologic deficit	- Peritumoral edema	/	/
**Prognostic factors related to epileptological outcome**	- Postresection ischemia and recurrent disease: independent predictors of worse seizure outcome. - WHO grade I tumor status: independent categorical predictor of improved Engel class outcome. - MIB-1 index: independent linear predictor of Engel class outcome.	- Tumor volume, sensomotory deficit and sphenoid wing location: independently associated with the occurrence of post-operative seizures. - Post-operative status epilepticus: independently associated with pre-operative seizures, sphenoid wing location and tumor volume.	- Peritumoral edema, high WHO grade, lower EOR: independent predictors of worse epileptological outcome.	- Pre-operative seizures, larger tumor size and continuation of ASMs: significantly associated with late post-operative seizures.	- Tumors located on left: predictor of post-operative seizures, - no post-operative complications and tumor location in the convexity/parasagittal/falx: lower risks of developing post-operative seizures.	- Younger age, tumor progression, and pre-operative epilepsy: predictors of post-operative epilepsy. - Risk factors for acute symptomatic seizures were radiographic gross total resection and surgical complications.	- Tumor progression and new permanent post-operative neurological deficit: significantly associated with late post-operative seizure.

* excluded 118 patients with multiple meningiomatosis.

## 5. Conclusions

Meningiomas are the most common non-malignant primary brain tumor and often associated with seizures. Seizure control is an important treatment goal because of the significant impact on patients’ quality of life. We identified age, convexity location, and peritumoral edema as the predictive risk factors of pre-operative seizures. In our experience, meningioma-related epilepsy appears to have generally a positive outcome following surgery and does not seem to be linked to oncological progression, even if further studies are needed.

Understanding seizure rates, risk factors, and possible mechanisms associated with meningioma-related epilepsy is crucial to better stratify and predict surgical outcome and optimally treat patients with the most effective treatment strategies.

## Figures and Tables

**Table 1:** Demographic, clinical, radiological, histological and molecular data of all study patients.

**Variables**	
**No. of patients**	358
**Gender**, *n* (%) Male Female	115 (32.1) 243 (67.9)
**Age**, (years) Mean (±SD) Range Age subclasses, *n* (%) 20–29 y 30–39 y 40–49 y 50–59 y 60–69 y 70–79 y ≥80 y	62.1 (±13.1) 23–90 2 (0.5) 16 (4.5) 46 (12.8) 82 (22.9) 96 (26.8) 92 (25.7) 24 (6.8)
**Pre-operative KPS, Mean (±SD)**	92.8 (20–100)
**Onset symptoms**, *n* (%) Seizures Other neurological symptoms No symptoms (incidental meningiomas)	73 (20.4) 226 (63.1) 59 (16.5)
**Tumor Side**, *n* (%) Right Left Midline	159 (44.4) 160 (44.7) 39 (10.9)
**Tumor Location**, *n* (%) Skull base Non-skull base (convexity)	148 (41.3) 210 (58.7)
**Pre-operative tumor volume (T1-weighted MRI images–cm^3^)** Mean Range	20.7 1–128
**Pre-operative peritumoral edema volume (T2-weighted MRI images–cm^3^)** Mean Range	22.9 1–227
**Type of Resection**, *n* (%) Gross Total Resection (I, II) Subtotal Resection (III, IV)	283 (79.1) 75 (20.9)
**Histological Grade**, *n* (%) WHO grade I WHO grade II WHO grade III	302 (84.4) 50 (13.9) 6 (1.7)
**Molecular aspects** Ki67, mean (±SD) Mitotic index, mean (±SD) Brain Invasion, *n* (%)	4.7 (±6.2) 1.81 (±4.8) 23 (6.5)
**Post-operative radiotherapy**, *n* (%) Yes No	12 (3.3) 346 (96.7)
**Post-operative neurological deficit 1 week after surgery**, *n* (%) Yes No	113 (32) 245 (68)
**Post-operative neurological deficit at 6 months**, *n* (%) Yes No	41 (11.9) 317 (88.1)

Legend: KPS, Karnofsky Performance Status; MRI, Magnetic Resonance Imaging; SD, Standard Deviation; WHO, World Health Organization.

**Table 2:** Characteristics of patients with meningioma-related epilepsy.

**Variables**	
**No. of patients**	73
**Gender**, *n* (%) Male Female	30 (41.1) 43 (58.9)
**Age**, (years) Mean (±SD) Range Age subclasses, *n* (%) 20–29 y 30–39 y 40–49 y 50–59 y 60–69 y 70–79 y >80 y	56 (±10.1) 39–87 0 (0) 6 (8.2) 13 (17.8) 22 (30.1) 19 (26.1) 10 (13.7) 3 (4.1)
**Seizure frequency**, *n* (%) Single Multiple	45 (61.6) 28 (38.4)
**Seizure onset**, *n* (%) Focal seizures Focal to bilateral tonic–clonic seizures	28 (38.4) 45 (61.6)
**Seizure type**, *n* (%) Motor Non-motor Somato-sensitive Cognitive Autonomic Affective	58 (79.5) 15 (20.5) 10 (13.7) 2 (2.7) 2 (2.7) 1 (1.4)
**Pre-operative EEG features**, *n* (%) Normal activity Slow activity Epileptiform activity	36 (49.4) 21 (28.7) 16 (21.9)
**ASMs regimen**, *n* (%) Monotherapy Polytherapy	67 (91.7) 6 (8.3)
**ASMs**, *n* (%) Levetiracetam Carbamazepine Valproic acid Perampanel	55 (82.1) 7 (10.4) 5 (7.5) 6 (8.3)

Legend: ASM, anti-seizure medication; EEG, electroencephalogram.

**Table 3:** Comparative analysis between patients with seizures and those without seizures at onset.

Variables	Onset without Seizures	Onset with Seizures	*p*-Value
	*n* = 285	*n* = 73	
**Age**, median (IQR)	66 (53–74)	56 (49–67)	**0.001**
**Female**, *n* (%) **Male**, *n* (%)	200 (70.2) 85 (29.8)	43 (58.9) 30 (41.1)	0.07
**Pre-operative KPS**, median (IQR)	100 (90–100)	90 (90–100)	**0.002**
**Post-operative KPS at 6 months**, median (IQR)	100 (90–100)	100 (90–100)	0.89
**Tumor Side**, *n* (%) Right Left Midline	123 (43.5) 129 (45.6) 31 (10.9)	36 (49.3) 31 (42.5) 6 (8.2)	0.61
**Tumor Location,** *n* (%) Non-skull base (convexity) Skull base	157 (55.1) 128 (44.9)	53 (72.6) 20 (27.4)	**<0.001**
**Simpson Grade**, *n* (%) Grade 1–2 Grade 3–4	218 (76.5) 67 (23.5)	65 (89.0) 8 (11.0)	**0.02**
**Pre-operative neurological deficit,** *n* (%)	152 (53.3)	27 (37.0)	**0.01**
**Post-operative neurological deficit (6 months),** *n* (%)	83 (32.1)	21 (28.7)	0.91
**Histological grade,** *n* (%) WHO grade I WHO grade II WHO grade III	246 (86.3) 33 (11.6) 6 (2.1)	56 (76.7) 16 (21.9) 1 (1.4)	0.06
**Molecular aspects** Ki67, median (IQR) Mitotic index, median (IQR) Brain invasion, *n* (%)	3 (2–5) 1 (0–2) 17 (6.0)	3 (2–5) 1 (0–2) 6 (8.2)	0.82 0.59 0.43
**Pre-operative tumor volume,** median (T1) (IQR)	20.6 (9.1–39.7)	20.8 (9.1–45.3)	0.70
**Peri-tumoral Edema** (T2), *n* (%)	122/271 (42.8)	56/71 (76.7)	**<0.001**
**Disease progression**, *n* (%)	23 (8.1)	6 (8.2)	0.97

Legend: KPS, Karnofsky Performance Status; IQR, interquartile range; WHO, World Health Organization. Bold values are statistically significant.

**Table 4:** Risk factors associated with pre-operative seizures.

Variable	Univariate Analysis			Multivariate Analysis		
	OR	95% CI	*p*-Value	OR	95% CI	*p*-Value
**Female**	0.61	0.36–1.04	0.067			
**Age**	0.97	0.95–0.99	**0.003**	0.95	0.93–0.97	**<0.001**
**Pre-operative KPS**	0.98	0.96–1.00	0.109			
**Tumor Side** Left vs. Right	0.82	0.48, 1.41	0.474			
**Tumor Location** Skull-base vs. convexity MCF vs. PCF	0.06 29.57	0.01–0.48 3.44–254.08	**0.007** **0.002**	0.08 17.05	0.01–0.62 1.86–155.94	**0.016** **0.012**
**Simpson Grade** 3 + 4 vs. 1 + 2	0.40	0.18–0.88	**0.022**			
**WHO Grade** Grade II vs. Grade I	2.17	1.12–4.22	**0.022**			
**Pre-operative tumor volume**	1.00	0.99–1.01	0.814			
**Pre-operative peritumoral Edema**	4.56	2.46–8.46	**<0.001**	5.28	2.62–10.61	**<0.001**
**Pre-operative neurological deficit**	0.51	0.30–0.87	**0.014**	0.75	0.42–1.37	0.352

Legend: CI, confidential interval; KPS, Karnofsky Performance Status; MCF, middle cranial fossa; OR, odds ratio; PCF, posterior cranial fossa; WHO, World Health Organization. Bold values are statistically significant.

**Table 5:** Seizure outcome in patients with pre-operative meningioma-related epilepsy at 12- and 24-months follow-up.

Variables	At 12 Months			At 24 Months		
	Engel Class Ia (*n* = 64)	Engel Class > Ia (*n* = 9)	*p*-Value	Engel Class Ia (*n* = 60)	Engel Class > Ia (*n* = 8)	*p*-Value
**Female,** *n* (%) **Male**, *n* (%)	38 (59.3) 26 (40.7)	5 (55.5) 4 (44.5)	0.96	36 (60.0) 24 (40.0)	5 (62.5) 3 (37.5)	0.98
**Age**, median (IQR)	57 (48–68)	56 (50–66)	0.77	56 (47.5–66.5)	56 (50–69)	0.79
**Tumor Side**, *n* (%) Right Left Midline	33 (51.5) 27 (42.3) 4 (6.2)	4 (44.4) 2 (22.2) 3 (33.3)	0.05	30 (50.0) 27 (45.0) 3 (5.0)	4 (50.0) 2 (25.0) 2 (25.0)	0.05
**Tumor Location** Non-skull base (Convexity) Skull base	48 (75.0) 16 (25.0)	4 (44.4) 5 (55.6)	**0.04**	45 (75.0) 15 (25.0)	4 (50.0) 4 (50.0)	**0.04**
**Simpson Grade** Grade 1–2 Grade 3–4	59 (92.2) 5 (7.8)	6 (66.7) 3 (33.3)	0.06	55 (91.7) 5 (8.3)	6 (75.0) 2 (25.0)	0.06
**Pre-operative tumor volume**, median (IQR)	21.4 (9.1–34.9)	25.1 (10.2–63.3)	0.66	20.8 (9.0–44.8)	33.5 (15.0–45.4)	0.35
**Seizure type**, *n* (%) Focal seizures Focal to bilateral tonic-clonic seizures	25 (39.1) 39 (60.9)	4 (44.4) 5 (55.6)	1.00	23 (38.3) 37 (61.7)	3 (37.5) 5 (62.5)	1.00
**Seizure frequency**, *n* (%) Single seizure Multiple seizures	40 (62.5) 24 (37.5)	4 (44.4) 5 (55.6)	0.30	37 (61.7) 23 (38.3)	5 (62.5) 3 (37.5)	1.00
**Motor seizures**, *n* (%)	52 (81.2)	6 (66.7)	0.39	48 (80.0)	5 (62.5)	0.36
**ASMs regimen**, *n* (%) Monotherapy Polytherapy	63 (98.4) 1 (1.6%)	7 (77.8) 2 (22.2)	**0.03**	59 (98.3) 1 (1.7)	1 (12.5) 7 (87.5)	**0.04**

Legend: ASM, anti-seizure medication; IQR, interquartile range. Bold values are statistically significant.

## Data Availability

All authors had full access to all the data in the study and take responsibility for the integrity of the data and the accuracy of the data analysis.
